# Relationship of Serum Leptin and Reproductive Hormones in Unexplained Infertile and Fertile Females

**DOI:** 10.7759/cureus.6524

**Published:** 2019-12-31

**Authors:** Mukhtiar Baig, Abid Azhar, Rehana Rehman, Hareem Syed, Saba Tariq, Zohair J Gazzaz

**Affiliations:** 1 Medical Education and Simulation, King Abdulaziz University, Jeddah, SAU; 2 Karachi Institute of Biotechnology and Genetic Engineering, University of Karachi, Karachi, PAK; 3 Biological and Biomedical Sciences, Aga Khan University, Karachi, PAK; 4 Internal Medicine, Civil Hospital Karachi, Dow University of Health Sciences, Karachi, PAK; 5 Pharmacology and Therapeutics, The University of Faisalabad, Faisalabad, PAK; 6 Internal Medicine, King Abdulaziz University, Jeddah, SAU

**Keywords:** unexplained infertility, leptin, body mass index, females

## Abstract

Objective: To investigate the relationship between serum leptin and reproductive hormones in females with unexplained infertility (UI).

Methodology: It was a case-control study conducted in the Gynecology and Obstetrics Department and Infertility Clinic, Jinnah Postgraduate Medical Center (JPMC), Karachi, Pakistan. A total of 235 primary infertile females with an unidentified cause of infertility were selected from the Infertility Clinics. The patients were excluded if they were found to have polycystic ovary syndrome, endometriosis, tubal blockage, irregular menstrual cycles, hyperthyroidism, hypothyroidism, hyperprolactinemia, hyperandrogenemia, fasting blood sugar >110 mg/dl, and male factor infertility. A total of 205 healthy, fertile females were selected from the general population. The blood samples of both groups were collected on the 12th and 21st day of their menstrual cycle. Serum leptin, follicle-stimulating hormone (FSH), luteinizing hormone (LH), and E2 levels were measured. Statistical analysis was executed using the SPSS version 16 (SPSS Inc., Chicago, IL).

Results: No significant difference was observed in leptin values of fertile and UI females, 37.110±1.19 vs. 35.321±0.901. In the preovulatory phase (12th day) of the cycle, infertile subjects with body mass index (BMI) <20 and 20-24.9 had significantly higher values for leptin (p<0.05), whereas, with an increase in BMI, leptin levels were reduced in these females. Leptin was reduced further in the luteal phase of infertile females with BMI 25-30, with a significantly lower value for FSH (p<0.005), LH (p<0.005), and estradiol (p<0.005. In infertile subjects, it correlated with estradiol (r=0.501, p<0.005), BMI (r=0.903, p<0.001), and progesterone (r=0.146, p<0.05).

Conclusion: Low levels of leptin observed to have an increase in the BMI of UI females were associated with a reduced estradiol and progesterone production in the luteal phase of the cycle.

## Introduction

Infertility is the inability of a sexually active couple, not using any contraceptive techniques in a year or more, to get pregnant [1]. Infertile couples suffer from trauma and depression when they experience delays, challenges, and disappointments in achieving reproduction. There are many types and causes of infertility; among which unexplained infertility (UI) is the only cause in 25% of couples reporting to assisted reproductive clinics. In fact, there are several factors which, when added, contribute to the presentation of UI; some of those factors include reduced sperm count and motility (within normal limits) and alterations in the time of follicle development and ovulation [2]. Leptin, a product of the ob gene which is primarily produced by adipose tissues, helps to regulate the hypothalamic-pituitary-gonadal axis and plays a vital role in obesity, hyperinsulinemia, and increased insulin resistance in the polycystic ovary syndrome (PCOS) patients [3]. An association of leptin with obesity, infertility, and other endocrine functions has been reported [4]. Circulatory leptin levels are considered to be a predictor of menstrual function. However, the regulatory loop by which leptin concentrations vary during the menstrual cycle is so far, not clear [5]. Leptin does not only modify gonadotropin-releasing hormone and gonadotrophin production, but it also plays an important role in the functioning of the ovary and endometrium and takes part in the development of an embryo [6]. 

It seems that the central and peripheral signals of the hypothalamic-pituitary-gonadal axis play an important role in the action of leptin in the menstrual cycle. Although the physiological impact of the alteration in the plasma leptin during the menstrual cycle is uncertain, it emerges that the lowest threshold of the circulating leptin is essential for a normal ovulatory function [7]. Therefore, leptin clearly appears to be linked to the reproductive system, probably coupling information regarding the adequacy of body energy homeostasis to the prospective event of pregnancy, demonstrating the importance of nutritional resources to a successful pregnancy. A study showed no significant alterations in the serum leptin concentrations, while alterations were observed in the fat mass or caloric intake in the obese and aged subjects, which reflects that peripheral signals released from adipocytes are not important in this group [8].

Higher leptin levels are considered to be the cause of infertility [9]. On the other hand, leptin stimulates the release of gonadotropins, follicle-stimulating hormone (FSH) and luteinizing hormone (LH), from the pituitary glands, and its administration has resulted in the resumption of fertility in experimental animals [10]. The leptin receptors have been discovered on gonads, which show that leptin may have a direct impact on the ovaries in females [11]. We, thus, wanted to determine leptin levels in females with UI in our population, and their variation with fertile females in the follicular and luteal phases of the menstrual cycle from non-obese to obese females. The present study was designed to compare the serum leptin and reproductive hormones in females with UI and fertile females.

## Materials and methods

The present study was carried out at the Department of Biochemistry, University of Karachi, Karachi, in collaboration with the Atomic Energy Medical Centre, Jinnah Postgraduate Medical Centre (JPMC), Karachi.

A total of 235 primary infertile female subjects were selected from the Infertility Clinic, JPMC, Karachi, Civil Hospital Karachi, Sobhraj Maternity Center Karachi, and Infertility Consultants. A total of 205 healthy female subjects were included in the study and were selected from the general population. The sample size was calculated using Open Epi software. It estimated 220 infertile females based on a 5% level of significance and 80% power of the study with a mean difference of serum leptin levels of -1.5 among fertile and infertile females. The approval for this research was granted by the Board of Advanced Studies and Research (No. BASR/9728/Sc), University of Karachi, Karachi, Pakistan, and subjects' anonymity was maintained, and strictly followed the Declaration of Helsinki. For this research, spanning a period of two and a half years, more than 1,000 infertile couples were interviewed and after taking their history and thorough investigations, 235 subjects were selected. Those subjects that were not fulfilling the inclusion criteria, due to the involvement of the male factor, PCOS, endometriosis, tubal blockage, irregular menstrual cycles, hyperthyroidism, hypothyroidism, hyperprolactinemia, hyperandrogenaemia, and fasting blood sugar >100 mg/dl, were excluded from the study. The selected women had no endocrine syndromes, and their husbands had proper parameters of sperm. Only those primary infertile female subjects, whose husbands were neither azoospermic nor oligospermic, that is, with sperm motility >70%, were selected. The blood samples were collected on the 12th and 21st day of their menstrual cycle. The inclusion criteria for infertile subjects were being between 20 and 40 years of age, primary infertile female, no uterine or pelvic pathology, no known cause of infertility, females having a regular menstrual cycle, and her husband having normal sperm parameters (normospermic and sperm motility >70%). We excluded the infertile subjects if they were suffering from PCOS, hirsutism, blocked ovarian tube, thyroid disease, being a diabetic patient, suffering from hyperprolactinemia, oligospermic or azoospermic husband, and husband’s sperm motility <50%. Their blood pressures were measured to check if they had a history of the disease, or any other disease, such as sexually transmitted diseases, hypertension, tuberculosis, and so on.

The inclusion criteria for fertile subjects were being female and having one or more children (control group), age ranging from 20 to 40 years, no uterine or pelvic pathology, and females having a regular menstrual cycle. The exclusion criteria for fertile subjects included diabetes, PCOS, hirsutism, thyroid disease, galactorrhea (hyperprolactinemia), pregnancy, lactation, and irregular and prolonged menstrual cycle.

All selected infertile female subjects and control fertile female subjects were grouped according to their body mass index (BMI) and were further grouped according to their phases of the menstrual cycle, that is, follicular and luteal phases. Group A included infertile subjects having BMI <20, group B included infertile subjects having BMI <25 (20-24.9), group C included infertile subjects having BMI <30 (25-29.9), and group D included infertile subjects having BMI >30.

The general and physical examination of subjects was done by measuring the height, weight, and hip and waist circumference of subjects, in order to determine the BMI and waist/hip (W/H) ratio. Subjects were weighed in light clothing and height was recorded, along with waist circumference measured at the level of the umbilicus and the hip circumference measured at the level of the greater trochanter. BMI (weight in kilogram/height in square meter) and W/H ratio was calculated using the formula. To characterize both groups of women, we gathered detailed information concerning their age, social background, jobs, and the length of time they have been trying to conceive. After obtaining written consent from the patients and normal subjects, 10 ml of blood was drawn by venipuncture from each subject in the follicular phase and 5 ml in the luteal phase of the menstrual cycle, the morning after an overnight fast (12 to 14 hours) using disposable plastic syringe under aseptic measures.

The blood was collected in a test tube and centrifuged for the separation of serum within an hour of blood collection, and then, the serum was stored in a deep freezer at a temperature of 70^o^C for subsequent analysis. Samples were analyzed in the batches of a 100 to omit in between analytical variations. Before analysis, samples were allowed to attain room temperature.

Serum estradiol, progesterone, FSH, LH, testosterone, T4, T3, TSH, and prolactin were measured by radioimmunoassay, using kits supplied by the Diagnostic System Laboratories (DSL Inc., Webster, TX). Immunoradiometric kit (DSL) was used for assaying serum leptin levels. The sensitivity of leptin IRMA kit (lowest detectable limit) was 0.1 ng/ml, and the intra- and interassay coefficients of variations were less than 8%. Glucose determination was done immediately by the enzyme oxidase method.

## Results

A total of 235 primary infertile (UI) female subjects and 205 healthy female subjects were included in the study. All selected infertile female subjects and control fertile female subjects were categorized according to their BMI and were further grouped according to their phases of the menstrual cycle, that is, preovulatory and luteal phases. No significant difference in serum leptin level was observed in both fertile and UI females, 37.110 ± 1.19 vs. 35.321 ± 0.901 ng/ml.

Table 1 shows leptin levels with respect to BMI in infertile and UI females. In the preovulatory phase (12th day) of the cycle, infertile subjects with BMI <20 and 20-24.9 had significantly higher values for leptin (p<0.05) as compared to the fertile ones. Decreased leptin levels were observed for females with BMI ranging between 25-29.9 and >30. In the luteal phase (21st day) of the subjects, UI females with BMI 25-29.9 showed decreased leptin levels. In the luteal phase, significant high leptin was observed in UI overweight and obese females. In other words, during the luteal phase, leptin rises in infertile females with a BMI between 25 and 30. Significantly higher levels of serum leptin (p<0.05) were found in the UI group, as compared to the fertile group in the preovulatory phase of the menstrual cycle, while no difference was found in the luteal phase. In the preovulatory phase, estradiol was significantly lower in UI subjects as compared to the fertile control group (p<0.005). 

**Table 1:** Comparison between serum leptin levels (ng/ml) in preovulatory and luteal phases, according to BMI in the fertile and UI females. Results are shown by mean±SEM. *p<0.05, **p<0.001. BMI, body mass index; UI, unexplained infertility.

Menstrual cycle phase	BMI <20		BMI 20-24.9		BMI 25-29.9		BMI >30	
	Fertile (n=27)	Infertile (n=38)	Fertile (n=66)	Infertile (n=66)	Fertile (n=64)	Infertile (n=72)	Fertile (n=47)	Infertile (n=51)
Preovulatory phase (12th day)	20.67±0.44	22.26±0.67*	26.29±0.55	27.54±0.34*	38.17±1.12	35.57±0.71*	61.20±2.20	55.90±1.80
Luteal phase (21st day)	22.28±0.43	23.19±0.65	28.19±56	28.56±0.36	40.18±1.10	37.21±0.73*	64.23±2.30	57.71±1.93*

Table 2 shows low E2 in infertile females in all BMI groups, except for BMI <20, whereas FSH and LH were low in the UI groups irrespective of BMI in the preovulatory phase. 

**Table 2:** Comparison between hormones of reproduction in the preovulatory phase in both fertile and UI females. Results are shown by mean±SEM. *p<0.05, **p<0.001. BMI, body mass index; FSH, follicle-stimulating hormone; LH, luteinizing hormone; UI, unexplained infertility.

Parameters	BMI <20		BMI 20-24.9		BMI 25-29.9		BMI >30	
	Fertile (n=27)	Infertile (n=38)	Fertile (n=66)	Infertile (n=66)	Fertile (n=64)	Infertile (n=72)	Fertile (n=47)	Infertile (n=51)
FSH (IU/L)	10.23±0.47	9.16±0.36*	10.37±0.33	2.88±0.11**	10.30±0.26	3.09±0.09**	10.21±0.33	2.91±0.09**
LH (IU/L)	25.01±0.89	22.28±1.31*	26.02±0.94	6.73±0.37**	24.50±0.70	6.68±0.3**	26.09±0.57	6.72±0.3**
Est (pg/ml)	208.45±6.11	212.61±9.68*	212.32±4.71	97.72±2.61**	264.40±6.11	122.72±3.21**	331.0±7.71	151.40±3.41**
Prog (ng/ml)	0.26±0.03	0.31±0.03*	0.34±0.03	15.73±0.19**	0.35±0.03	15.73±0.19**	0.37±0.04	15.63±0.22**

In the luteal phase (21st day) of their menstrual cycle, significantly lower values for FSH (p<0.005), LH (p<0.005), and estradiol (p<0.005) were found in the UI subjects when compared with those of the control subjects. Comparison by BMI showed a significantly low FSH, LH, and E2 in infertile females of all BMI groups (Table 3).

**Table 3:** Comparison between hormones of reproduction in the luteal phase in both fertile and UI females Results are shown by mean±SEM. *p<0.05, **p<0.001. BMI, body mass index; FSH, follicle-stimulating hormone; LH, luteinizing hormone; UI, unexplained infertility.

Parameters	BMI <20		BMI 20-24.9		BMI 25-29.9		BMI >30	
	Fertile (n=27)	Infertile (n=38)	Fertile (n=66)	Infertile (n=66)	Fertile (n=64)	Infertile (n=72)	Fertile (n=47)	Infertile (n=51)
FSH (IU/L)	2.79±0.14	2.13±0.094**	10.34±0.302	2.33±0.067*	9.91±0.310	2.26±0.070**	10.30±0.36	2.38±0.094**
LH (IU/L)	5.96±0.41	3.45±0.21**	24.55±1.00	3.48±0.14*	21.49±1.99	3.80±0.20**	22.70±1.70	3.66±0.24**
Est (pg/ml)	97.62±3.71	87.61±2.00*	206.11±6.11	92.20±2.30	220. 61±5.81**	102.02±2.70**	296.11±8.90*	116.10±3.31**
Prog (ng/ml)	16.44±0.31	15.91±0.10	0.31±0.03	16.02±0.17	0.37±0.035	16.06±0.35	0.41±0.04	16.56±0.24**

**Table 4:** Correlation coefficient (r) of serum leptin with hormones of reproduction in both fertile and UI females. *p<0.05, **p<0.001. BMI, body mass index; FSH, follicle-stimulating hormone; LH, luteinizing hormone; UI, unexplained infertility.

Parameters	Preovulatory phase		Luteal phase	
	Fertile control (n=204)	Infertile subjects (n=235)	Fertile control (n=204)	Infertile subjects (n=235)
FSH (IU/L)	0.033	0.045	0.057	0.045
LH (IU/L)	0.112	-0.040	0.113	0.035
Est (pg/ml)	0.777*	0.589*	0.716**	0.501**
Prog (ng/ml)	0.079	0.054	-0.039	0.146*
BMI (kg/m^2^)	0.905*	0.903*	0.911**	0.903**

In the preovulatory phase (12th day) of the menstrual cycle, a significant correlation of serum leptin was observed with estradiol (r=0.777, p<0.01) and the BMI (r=0.905, p<0.01) in the fertile group, whereas in the infertile group, the serum leptin level was correlated with estradiol (r=0.589, p<0.001) and BMI (r=0.903, p<0.01). In the luteal phase (21st day) of the menstrual cycle, a significant correlation of serum leptin was found with estradiol (r=0.716, p<0.001) and BMI (r=0.911, p<0.001) in the fertile subjects, whereas in the UI subjects, it correlated with estradiol (r=0.501, p<0.005), BMI (r=0.903, p<0.001), and progesterone (r=0.146, p<0.05) (Table 4).

## Discussion

The dependence of the reproductive function on energy resources, body weight, body composition, and fat distribution has been documented. Leptin is an adipose tissue-derived messenger of the amount of energy stores in the brain, which acts as a crucial hormone/cytokine that controls some functions, such as reproduction [12]. It is observed that decreased leptin levels disrupt the neuroendocrine regulation of reproduction [13]. At the same time, high leptin levels are likely to exert a negative influence on the normal ovarian function and on fertilization that is required for the development of the embryo [14]. We tried to evaluate whether leptin exhibits a dual role or not in the regulation of reproduction and whether it is the same for females with different BMIs.

High serum leptin level was observed by a few researchers in infertile women [9,15]. In our study, the overall leptin levels in fertile and infertile females did not differ significantly. The difference in results could be due to the different genetic makeup, different sample sizes, and differences in BMI. However, in the BMI groups <20 and between 20 and 24.9 (low to normal), significantly higher levels of leptin were observed in the UI females during the preovulatory phase. Leptin plays an important role in follicular development and consequent luteal function [12]. The hormones of reproduction were compared in both phases of the female cycle (follicular and luteal phases) to confirm the role of leptin in UI. The study is comparable with that of Demir et al., who reported significantly higher levels of leptin in the early follicular phase (day 3) of the menstrual cycle, in all BMI-matched females with UI [15].

An interesting finding in our study was that the leptin levels were elevated during the luteal phase of the menstrual cycle, and they were significantly higher in UI females with BMI <20 and between 20 and 24.9 (low BMI). Wunder et al. have reported an increase in circulating leptin levels from the follicular to the luteal phase [16]. Another study by Ajala et al. concluded that the leptin levels are highest in the luteal phase of the menstrual cycle [17]. The serum leptin level was significantly higher in the luteal phase of normal weight UI subjects, but there was a significant fall in the leptin levels of UI overweight and obese females during this phase. It appears that the BMI above normal level influences the relationship between serum leptin and menstrual cycle, and obesity induces abnormal rhythmicity that has been hypothesized to contribute to leptin resistance in obesity [18]. The increase in serum leptin during the luteal phase of the cycle in both fertile and infertile women having normal weight was observed in our study that has been reported by others [19]. Farooq et al. observed the high leptin level in obese fertile and UI females, as compared to the normal fertile females, but their values were lesser than in normal UI females that were not observed in our study [20].

Change in leptin levels can be considered as one of the factors responsible for causing infertility, as a result of its impact on estradiol (E2) production by human granulosa cells, in response to LH [12]. An association of E2 with leptin has been demonstrated during the follicular phase of both spontaneous and exogenous stimulated menstrual cycles [21]. The low E2 in infertile females correlated with slightly raised leptin levels in low BMI females and reduced leptin levels in high BMI females of UI. The positive correlation between leptin and E2 indicates that leptin affects human ovary either directly or under the influence of the gonadal steroids. The hypothesis is supported by the fact that the treatment of human granulosa cells with leptin causes the inhibition of the FSH-stimulated E2 production [22].

A study by Farooq et al. reported that serum leptin has a strong negative correlation with LH, FSH, and testosterone in the fertile obese as well as in normal males and females, which were statistically significant [20]. This study, however, did not find any such correlation of leptin with FSH and LH. High E2 in the follicular as well as in the luteal phase is responsible for increasing fertility [1]. This finding raises the speculation that a low E2 production is responsible for UI due to high leptin levels in the normal, whereas leptin resistance may lead to a decreased E2 production in obese females. Serum E2 level correlated with serum leptin in UI overweight and obese groups with serum leptin in the fertile control group B (20-24.9), (25-29.9), and (>30) in both phases of the cycle, but the underweight UI groups did not show any correlation.

As serum E2 has a significant correlation with leptin in infertile subjects, it is likely that an increased fat mass may play some role in establishing a relationship between serum estradiol and serum leptin. Serum leptin levels of females with UI were similar to females with a PCOS, although a high leptin content was observed in the peritoneal fluid of the UI females [9]. The peritoneal and serum leptin levels in UI, however, were not different from that of females of endometriosis, measured in the late follicular phase [23]. Ovarian production of leptin has been shown to be probably caused by the preovulatory surge of estrogen that, in turn, stimulates the gonads to produce more leptin together with the escalating titers of progesterone. No change was observed in serum progesterone in both phases of the menstrual cycle in any group. 

Limitation of the study is the consideration of BMI as a measure of body fat storage that masks the underlying excess adiposity that can be depicted by an increased percentage of fat and a reduced muscle mass. The study should have compared serum and follicular leptin levels. At the same time, cases of UI may be due to the contributions from minimal factors from both partners, as the borderline semen analysis and poor ovarian responsiveness were not excluded from the study.

The increase in leptin levels during the luteal phase of the menstrual cycle and the occurrence of leptin receptors in the ovaries need to be further explored.

## Conclusions

An association of leptin and E2 levels in non-obese to obese females with UI was observed in our population. Low leptin levels can explain UI in obese females with reduced E2 and progesterone production, required for implantation of the fertilized ovum. 
